# REPIMPACT - a prospective longitudinal multisite study on the effects of repetitive head impacts in youth soccer

**DOI:** 10.1007/s11682-021-00484-x

**Published:** 2021-09-10

**Authors:** Inga K. Koerte, Roald Bahr, Peter Filipcik, Jolien Gooijers, Alexander Leemans, Alexander P. Lin, Yorghos Tripodis, Martha E. Shenton, Nir Sochen, Stephan P. Swinnen, Ofer Pasternak, for the REPIMPACT Consortium Investigators

**Affiliations:** 1 cBRAIN, Department of Child and Adolescent Psychiatry, Psychosomatics, and Psychotherapy, Ludwig-Maximilians-Universität, Munich, Germany; 2 Department of Psychiatry, Psychiatry Neuroimaging Laboratory, Brigham and Women’s Hospital, Harvard Medical School, Boston, MA USA; 3 Oslo Sports Trauma Research Center, Department of Sports Medicine, Norwegian School of Sport Sciences, Oslo, Norway; 4 Institute of Neuroimmunology, Slovak Academy of Sciences, Bratislava, Slovakia; 5 Movement Control and Neuroplasticity Research Group, Department of Movement Sciences, Goup Biomedical Sciences, KU Leuven, Leuven, Belgium; 6 KU Leuven Brain Institute (LBI), Leuven, Belgium; 7 PROVIDI Lab, Image Sciences Institute, University Medical Center Utrecht, Utrecht University, Utrecht, The Netherlands; 8 Center for Clinical Spectroscopy, Department of Radiology, Brigham and Women’s Hospital, Harvard Medical School, Boston, MA USA; 9 Department of Biostatistics, Boston University School of Public Health, Boston, MA USA; 10 Boston University Alzheimer’s Disease Center and Boston University CTE Center, Boston University School of Medicine, Boston, MA USA; 11 Department of Radiology, Brigham and Women’s Hospital, Harvard Medical School, Boston, MA USA; 12 Department of Applied Mathematics, School of Mathematical Sciences, Tel Aviv University, Tel Aviv, Israel; 13 Sagol School of Neuroscience, Tel Aviv University, Tel Aviv, Israel

**Keywords:** Repetitive head impacts, Youth athletes, Sport-related brain injury, Soccer

## Abstract

Repetitive head impacts (RHI) are common in youth athletes participating in contact sports. RHI differ from concussions; they are considered hits to the head that usually do not result in acute symptoms and are therefore also referred to as “subconcussive” head impacts. RHI occur e.g., when heading the ball or during contact with another player. Evidence suggests that exposure to RHI may have cumulative effects on brain structure and function. However, little is known about brain alterations associated with RHI, or about the risk factors that may lead to clinical or behavioral sequelae. REPIMPACT is a prospective longitudinal study of competitive youth soccer players and non-contact sport controls aged 14 to 16 years. The study aims to characterize consequences of exposure to RHI with regard to behavior (i.e., cognition, and motor function), clinical sequelae (i.e., psychiatric and neurological symptoms), brain structure, function, diffusion and biochemistry, as well as blood- and saliva-derived measures of molecular processes associated with exposure to RHI (e.g., circulating microRNAs, neuroproteins and cytokines). Here we present the structure of the REPIMPACT Consortium which consists of six teams of clinicians and scientists in six countries. We further provide detailed information on the specific aims and the design of the REPIMPACT study. The manuscript also describes the progress made in the study thus far. Finally, we discuss important challenges and approaches taken to overcome these challenges.

## Introduction

Repetitive head impacts (RHI) and concussions are common in athletes participating in contact sports. RHI differ from concussion as RHI are considered hits to the head that usually do not result in acute symptoms and are therefore also referred to as “subconcussive” head impacts. However, recent studies suggest that RHI, particularly when sustained in close proximity in time, may have cumulative effects (Koerte et al., 2012; Koerte, Lin, Muehlmann, et al., 2015a; Koerte, Lin, Willems, et al., 2015b; Koerte, Mayinger, Muehlmann, et al., 2015c). Nonetheless, little is known about brain alterations caused by RHI, or about the risk factors that lead to clinical or behavioral sequelae.

Soccer provides an accessible model to investigate the effects of RHI. Soccer is the most popular and fastest growing sport in the world with an estimated 265 million players (http://www.fifa.com2021, 2021). Soccer is unique in that the unprotected head is intentionally used when heading the ball, making it the sport with the largest number of RHI (Covassin et al., 2003a, 2003b; Gessel et al., 2007). Match heading frequency is greater for boys than girls, but increases with age (Sandmo, Andersen, et al., 2020a), and – with the addition of many more during practice – players are likely exposed to thousands of headings during their career. Head accelerations of up to 20 g may apply when heading a soccer ball (Naunheim et al., 2003; Sandmo, McIntosh, et al., 2019; Shewchenko et al., 2005a, 2005b, 2005c). Indeed, there is some evidence to suggest a link between RHI in soccer players and brain alterations (Koerte et al., 2012; Koerte, Lin, Muehlmann, et al., 2015a; Koerte, Mayinger, Muehlmann, et al., 2015c). More severe effects can be expected when a child or adolescent with insufficient neck strength or motor control attempts to head a high velocity ball (Covassin et al., 2012; Harmon et al., 2013; Hessen et al., 2007).

Recently, a directive by the United States Soccer Federation to ban headings for players under age 11 has been followed by The Football Association in England as well as by The Scottish Football Assocation (Association, 2020; England, 2020; Soccer, 2019). However, there is very limited scientific evidence in children 11 years or younger playing soccer to support such directives. On the other hand, there is preliminary evidence to suggest that RHI may have harmful effects on older youth players as well. For example, a small study on female youth soccer players (15–18 years) found cognitive dysfunction immediately after exposure to RHI during a soccer practice (number of performed headers based on self-report: median 6; range 2–20) (M. R. Zhang et al., 2013). Another study in 10 young adult soccer players (mean age 21 years) found transient dysfunction of vestibular processing one day following an experimental exposure to bouts of RHI (Hwang et al., 2017). Moreover, one study in male youth soccer players (15–17 years) showed less improvement on a cognitive task over the course of a play season, compared with table tennis players (Koerte et al., 2017). However, the above mentioned studies were small (*n* < 20) and did not employ quantitative measures such as neuroimaging or fluid biomarkers to investigate the underlying pathophysiology (for review see (Tarnutzer et al., 2017). Nonetheless, these studies support the hypothesis that exposure to RHI in youth athletes may disrupt brain processes and thus form the rationale for studying youth soccer players with an array of objective, quantitative measures.

The current paper describes the aims, study design, and methodological approach of the REPIMPACT study as well as the structure of the REPIMPACT Consortium. We also describe the progress made in the study, the characteristics of the study population as well as significant challenges and approaches taken to overcome these.

## Study aims

**Fig. 1:**
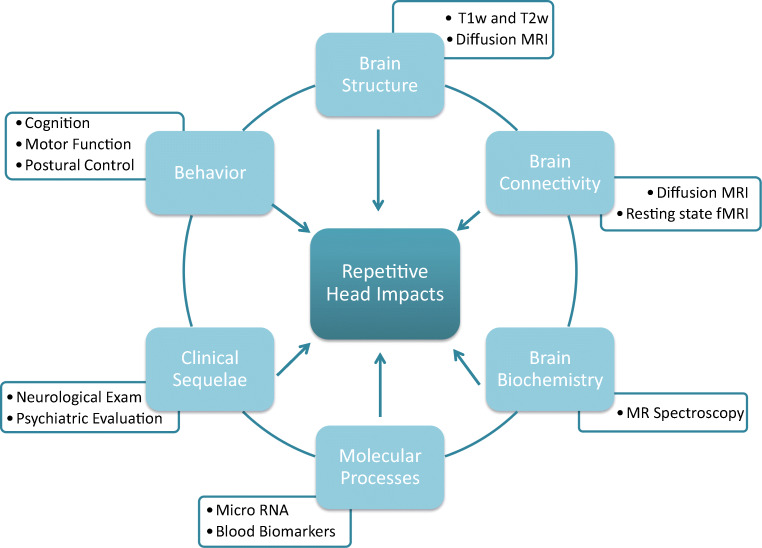
REPIMPACT aims to characterize between-group differences in behavior, clinical sequelae, molecular processes, as well as neuroimaging measures of brain biochemistry, brain connectivity, and brain structure

REPIMPACT is a prospective longitudinal multisite study of competitive youth soccer players and control athletes who do not participate in contact sports. The study aims to characterize between-group differences in behavior, clinical sequelae, neuroimaging measures, as well as blood- and saliva-derived measures of molecular processes, over the course of one year (Fig. 1). A second aim is to explore the association between RHI exposure and changes in outcome measures over time within the soccer group.

## Consortium structure

The REPIMPACT Consortium includes investigators from 6 research groups based in 6 countries: Germany, Belgium, Israel, Norway, Slovakia, and The Netherlands, as well as consultants from institutions in the U.S. Altogether, the REPIMPACT Consortium consists of physicians, neuroscientists, computer scientists, mathematicians, engineers, statisticians, computational biologists, psychologists, and neurobiologists. Collectively, the research team has expertise in traumatic brain injury, sports-related concussion, sports-medicine, neurology, child neurology, child psychiatry, advanced structural and functional neuroimaging, mathematical modelling, medical image processing, integrative computational biology, statistical analysis, neuropsychology, cognitive neuroscience, neurophysiology, neurodegeneration, and neuroimmunology.

The consortium included three data acquisition sites: Germany (Ludwig-Maximilians-Universität, Munich), Belgium (KU Leuven and University Hospitals Leuven, Leuven), and Norway (Oslo Sports Trauma Research Center, Oslo). The three additional sites contributed with specific expertise: Imaging protocols and algorithm development were headed by the group in Israel (Tel-Aviv University, Tel-Aviv). The group in The Netherlands (Utrecht University, Utrecht) led image post-processing. Fluid biomarker analyses were headed by the group in Slovakia (Institute of Neuroimmunology, Slovak Academy of Sciences, Bratislava). The consultants added specific expertise in MR spectroscopy, neuroimaging in traumatic brain injury, and biostatistics to the Consortium. All specific tasks were divided into work packages and distributed between the consortia partners.

## Approach

### Study design

REPIMPACT is a prospective longitudinal multisite study evaluating youth soccer players with exposure to RHI compared to a control group of athletes without exposure to RHI. The REPIMPACT study collected a comprehensive longitudinal battery of quantitative measures across three time points within a period of 15 months: TP1 – before the beginning of the competitive season, TP2 – towards the end of the season, TP3 – 2 months after TP2.

### Study participants

Youth athletes were recruited from competitive soccer clubs in Germany, Belgium, and Norway. We presented the study aims and provided information relevant to the study to players, parents, and coaches at competitive youth soccer clubs and academies in Germany, Belgium, and Norway. Control athletes without exposure to RHI were recruited through email and information relevant to the study that was addressed to relevant sports associations and clubs (e.g., swimming). Due to limited funding, only male athletes were included.

Inclusion criteria for soccer players were: 14–16 years of age, male, participation in competitive soccer with at least three soccer training sessions per week, and proficiency in the language of the respective country (i.e., German, Dutch, and Norwegian). Inclusion criteria for the control athletes were: 14–16 years of age, male, participation in a competitive non-contact sport with at least three weekly training sessions, no history of contact sports within 12 months prior to inclusion in the study, and proficiency in the language of the respective country (i.e., German, Dutch, and Norwegian). Exclusion criteria for both groups were: a history of physician-diagnosed concussion or any other form of traumatic brain injury, recent brain surgery, physician-diagnosed developmental or learning disorder, any active or past neurological disorder, history of prematurity (birth prior to 37 weeks of gestation), intake of neuroleptic or psychiatric medication, illegal substance abuse, and contraindications to magnetic resonance imaging (MRI).

### Study protocol

The study protocol at each time point (TP1-TP3) included collection of demographics, medical history, and previous exposure to RHI, comprehensive neuropsychological evaluation, neuropsychiatric screening, balance assessment, neuroimaging, and blood and saliva collection (Fig. 1). Most procedures were identical between data acquisition sites (Table 1), and those that were not identical are mentioned in the text below.

**Table 1:** Performed REPIMPACT study protocol at all data acquisition sites (GER = Germany, BEL = Belgium, NOR = Norway). The study protocol was consistent across the three data acquisition sites. The additional dynamic balance test was only acquired in Belgium because of the required specific equipment

	GER	BEL	NOR
MRI	+	+	+
MRS	+	+	+
Neurological examination	+	+	+
Balance test	+	+	+
Dynamic balance test	–	+	–
Neuropsychological testing	+	+	+
Neuropsychiatric screening	+	+	+
Blood collection	+	+	+
Saliva sampling	+	+	+
Questionnaire training habits	+	+	+
Intelligence test	+	–	–

#### Demographics, Medical History, and Exposure to RHI

We conducted a semi-structured interview to capture demographic data and information on current and previous sport history, concussion, and pre-existing personal and medical history. Participants also completed a standardized measure of self-reported symptoms from the Sport Concussion Assessment Tool 5 (SCAT5) symptom list. Detailed information was acquired on training habits and life-style, including age at start of systematic training, hours of training per week, number of headings performed per day/week/year, position in the field, injuries in general, head injuries, history of symptomatic concussion, and handedness. In addition, “HeadCount” (Lipton et al., 2013), a previously published standardized questionnaire, was validated for this age group and then applied to evaluate exposure of RHI. This questionnaire gathers information on intentional headers as well as on unintentional head impacts sustained in practices and games.

#### Intelligence

A measure of fluid intelligence (Culture Fair Intelligence Test (CFT20-R)) was administered at the first time point. IQ testing was performed at the German site only.

#### Neuropsychiatric Screening

In order to screen for psychiatric symptoms the Youth Self Report (YSR) (Achenbach & Rescorla, 2001) was administered to gather information about the following dimensions: anxious/depressed, withdrawn/depressed, somatic complaints, social problems, thought problems, attention problems, rule-breaking behavior, and aggressive behavior.

#### Neurological Evaluation

A semi-standardized neurological evaluation including neurological and developmental history as well as neurological examination that were performed to screen for coincidental and confounding neurological illnesses, and to document the presence or absence of neurological abnormalities. Whenever possible, the neurological exam was performed at TP1; however, for practical reasons, in some cases it was performed instead at TP2 or TP3. A neurological examination was performed to evaluate cranial nerves, motor strength and tone, deep tendon reflexes, sensation, coordination, fine motor function and gait, validated and standardized as described elsewhere (Hadders-Algra et al., 2010).

#### Neurocognitive functioning

Was evaluated through a computer assessment of psychomotor function, executive function, mental flexibility, processing speed, attention, learning, memory, and working memory using the “Cogstate” Computerized Cognitive Assessment Tool (Collie et al., 2007).

#### Balance Assessment

Balance performance was assessed using the Balance Tracking System (Goble et al., 2016) (BTrackS, Balance Tracking Systems Inc., San Diego, CA, USA) in all three data acquisition sites. The EquiTest System (NeuroCom International, Clackamas, OR, USA) was used in addition at the Belgian site.

#### Neuroimaging

REPIMPACT collected 3 T MRI data were acquired using a common protocol between the three data acquisition sites (Table 2). Sequences were designed to optimize sensitivity to alterations in brain structure and function, while minimizing site differences, allowing for better harmonization. Before data acquisition, each site was asked to scan an identical phantom, which was used to assure that the MRI scanners provide comparable signal and comparable image deformations. The three scanners were from the same vendor (Philips) and had the same major software version (5.3). Sequence parameters were matched across scanners to minimize between-site variability. Diffusion and functional MRI sequences were identical in Germany and Belgium and leveraged multi-band acquisitions. Since the Norwegian site did not have multi-band capabilities, matching sequences that do not use multi-band were designed and installed. Finally, test-retest experiments were performed on healthy volunteers at each site, to evaluate the comparability of within-site and between-site variability (“travelling heads”). To further assure site comparability, an imaging manual was provided for each site and the technicians in each site were instructed on how to follow this manual when acquiring the data by representatives from the REPIMPACT consortium. The imaging protocol included: 1) high-resolution T1-weighted and T2-weighted anatomical images to characterize regional and gross gray and white matter volume, cortical thickness, and myelin mapping; 2) a multi-shell diffusion acquisition with low and high-b-values to investigate brain tissue microstructure and structural connectivity. The multiple shells allow the application of novel analysis methods that go beyond the common DTI analysis such as free-water imaging (Pasternak et al., 2009), neurite orientation dispersion and density imaging (NODDI) (Zhang et al., 2012), and diffusion kurtosis imaging (DKI) (Jensen et al., 2005). Parameters of these models are putative measures for gliosis, vasogenic and cytotoxic edema, axonal and myelin pathology, atrophy, and neuroinflammation; 3) High spectral resolution and multiple regions-of-interest MR spectroscopy sequences to assess brain metabolism. Short echo single voxel spectroscopy was acquired in the anterior cingulate gyrus and the periventricular white matter, which have been characterized by abnormalities in TBI (Bartnik-Olson et al., 2020); 4) Functional MRI sequence to detect subtle alterations of functional connectivity at resting state complementing structural MRI and diffusion MRI (Fig. 3).

**Table 2:** Performed magnetic resonance imaging sequences including parameters

Sequence	Imaging technique	Flip angle [*]	Field of view [mm]	Voxel-size [mm^3^]	TE [ms]	TR [ms]	Acceleration	Extra notes
T1-weighted	3D gradient echo	8	256x256x180	1x1x1	3.7	8	SENSE 2 + 2.6	Inversion time 950 ms
T2-weighted	3D turbo spin echo	90	256x256x160	1x1x1	shortest (252)	2500	SENSE 2 + 1.5	refocusing 35^*^
FLAIR	3D inversion recovery	90^*^	240X240X179	1.12 × 1.12 × 1.12	shortest (288)	4800	SENSE 3 + 2	Inversion Time 1600 ms. T2 preparation (125 ms) + Refocusing 40^+^
dMRI	^2D^ spin echo EPI	90	224x224x160	2x2x2	113	7170 [12000]	MB 2 + SENSE 2 [SENSE 2.5]	97 [98] gradient directions over 13 b-values
rsfMRI	2D gradient echo EPI	80	228x228x143	2.75 × 2.75 × 2.75 [3x3x3]	27	1600 [2100]	MB 2 + SENCE 2 [SENSE 3]	300 dynamics

The values indicated within [] are specific for the sequence without multi-band

#### Fluid Biomarkers

Blood and saliva samples were collected, preprocessed, and stored at −80 °C before shipping the samples to Bratislava, Slovakia for further analyses. Blood-derived measures: The rationale for analyzing miRNAs is based on recent reports on their potential diagnostic value in traumatic brain injury (Atif and Hicks, 2019; Toffolo et al., 2019). Moreover, miRNAs may potentially provide insight in the processes (physiological and pathological) induced by repetitive head impacts. Because miRNAs cross the blood brain barrier and are stable in peripheral tissue and fluid, they can be quantified in e.g., plasma and saliva. In addition, specific neuro- and immune-related proteins such as total/phosphorylated microtubule associated tau protein, neurofilament light polypeptide, ubiquitin C-terminal hydrolase-L1, glial fibrillary acidic protein, and cytokines (IL6, IL10, TNFα) have recently been shown to serve as promising biomarkers and prognostic indicators of traumatic brain injury (Bhomia et al., 2016; Di Pietro et al., 2017; Gill et al., 2018; Peltz et al., 2020; Redell et al., 2010; Yang et al., 2016). Validated circulating miRNAs will be analyzed using integrative computational biology to identify dysregulated pathways associated with RHI. In a secondary and exploratory aim, we investigate the utility of the above listed measures extracted from saliva as possible replacement for blood samples.

#### Statistical approach

For the statistical analysis of the repeated measures, we will use linear mixed effect models. Our models will include a fixed effect for site, age of the participant (in months), time since baseline (in months), a group variable (soccer vs control) and a binary variable indicating whether the measurement is baseline (TP1) or during the course of the study (TP2 and TP3). We will also include fixed effects for time since baseline interactions with group and with the measurement occasion. Finally, we will also include a random intercept for each subject and a random slope for time since baseline to account for the between and within subject variability. Our main hypothesis is that there will be significant differences in the change of the outcomes between soccer players and controls over the course of the three time points; this will be tested using the parameter of baseline x group interaction. Other hypotheses are: 1) there are group differences at baseline (TP1), which will be tested using the parameter of the group main effect; and 2) there is an association between changes in outcome measures and exposure to RHI. To evaluate the association between RHI exposure and changes in outcome measures over time, the model will be applied to the soccer players only and instead of group as exposure variable, we will include measures of head impact exposure. To adjust for inflated false-positive due to multiple comparisons, we will apply the Benjamini-Hochberg procedure to restrict false discovery rate at 5% level. All analyses will be conducted in R (R Core Team, 2017) with lme4 (Bates et al., 2005).

## Progress

### Enrollment

Launched in the fall of 2017, the REPIMPACT Consortium has completed the planned data collection. The three data acquisition sites have successfully enrolled 129 athletes into the study. Monthly conference calls among the REPIMPACT Consortium have ensured consistency of enrollment criteria and data sampling between the three data acquisition sites.

**Fig. 2:**
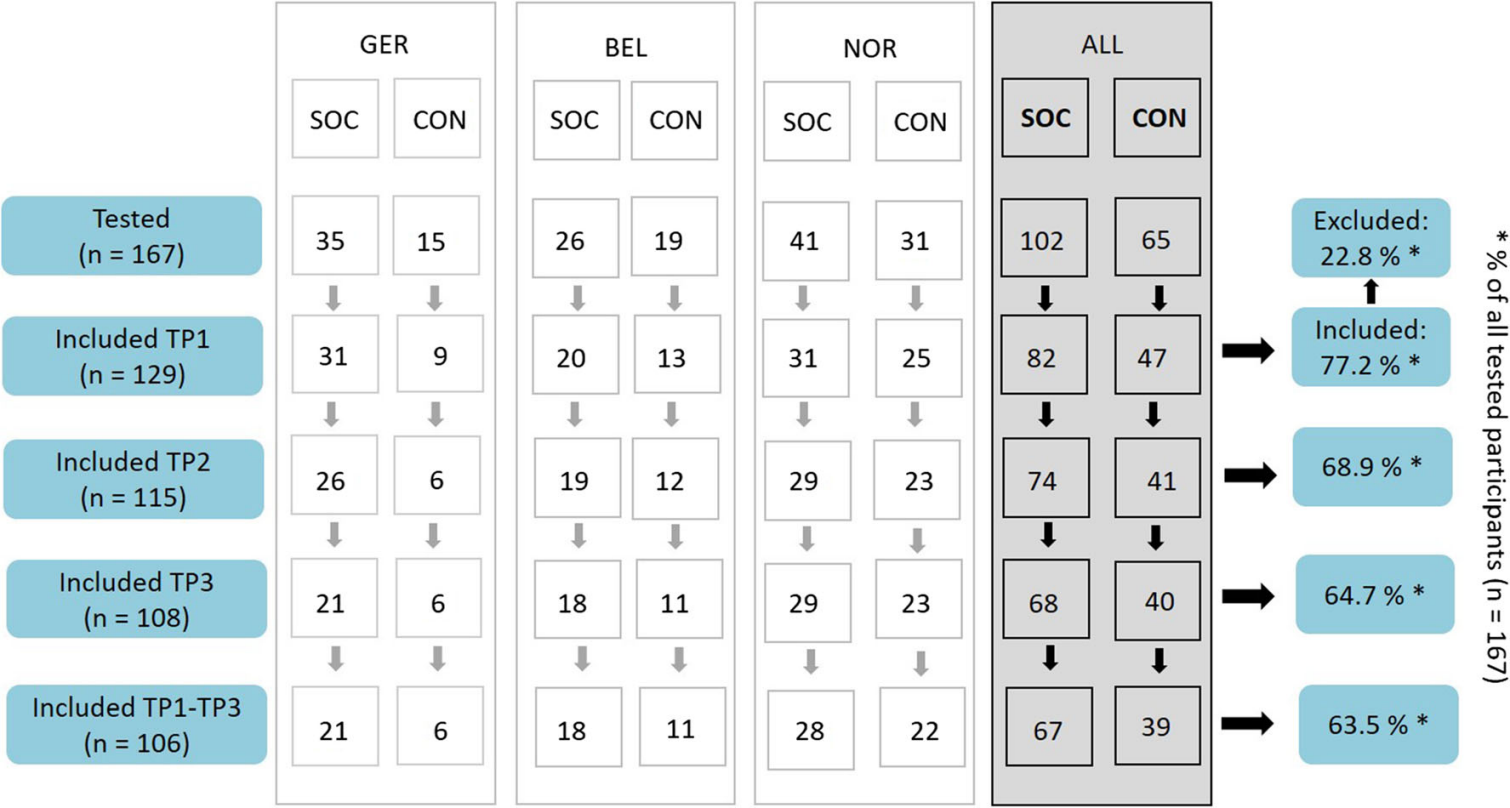
Overview of tested and included participants at the three timepoints at all data acquisition sites. *Abbreviations*. SOC = soccer players, CON = control athletes, GER = Germany, BEL = Belgium, NOR = Norway, TP1 = first assessment, TP2 = second assessment, TP3 = third assessment

In total, 167 athletes were invited for testing. Of those, 35 were excluded during the medical interview or neurological examination (history of physician-diagnosed concussion (*n* = 15), physician-diagnosed migraine (*n* = 2), history of prematurity (*n* = 4), dyslexia (*n* = 7), history of viral or cerebral infections (*n* = 3), oppositional defiant disorder (n = 1), congenital hydrocephalus (n = 1), claustrophobia (n = 1), control participant actively playing contact sport (n = 1)). In addition, three participants were excluded after inclusion due to an incidental finding on MRI suggestive of a neurological abnormality. Control athletes participated in a variety of non-contact sports (Table 3). The final cohort included a total of 82 soccer players and 47 control athletes (Fig. 2). Of these 129 athletes, 106 completed all three time points. The mean time between TP1 and TP2 was 9.6 months (range 6.9–14.0 months). The time between TP2 and TP3 was on average 2.7 months (range 0.5–6.0 months). The mean time between TP1 and TP3 was 12.2 months (range was 8.9–16.1 months). Of note, due to a nation-wide lockdown in the spring and summer of 2020, 3 of the enrolled controls and 4 of the enrolled soccer players could not be followed up at TP2 and TP3 in Germany.

**Table 3:** Sports performed by control athletes listed for each data acquisition site: Germany (GER), Belgium (BEL), Norway (NOR). A total of 47 athletes were included in the control group. The table lists a total of 56 sport participations due to participation of some athletes in two sports (1 athlete in Belgium and 7 athletes in Norway).

Types of sport	GER	BEL	NOR	ALL
Swimming	2	5	6	13
Track&Field/Athletics	2	4	0	6
Cross-country skiing	0	0	6	6
Cycling	0	0	5	5
Tennis	0	0	5	5
Biathlon	0	0	5	5
Rowing	2	1	0	3
Tabletennis	2	1	0	3
Orienteering	0	0	2	2
Triathlon	0	1	1	2
Badminton	0	1	0	1
Kayaking	0	0	1	1
Rollerskating	0	1	0	1
Volleyball	0	0	1	1
Gymnastics	1	0	0	1

### Neuroimaging

**Fig. 3:**
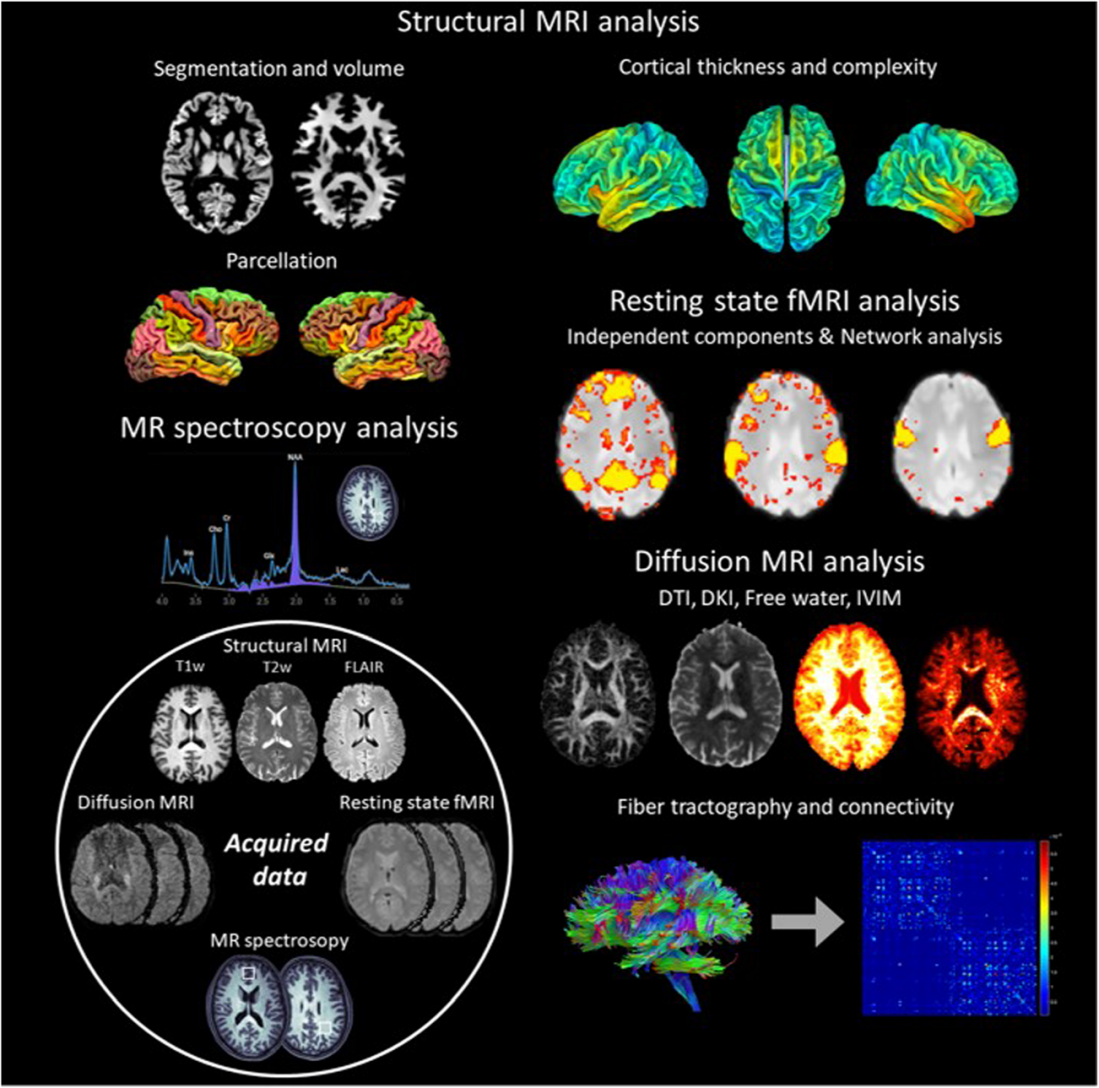
The REPIMPACT imaging protocol includes sequences for the acquisition of structural, diffusion and function MRI data. Sequences were designed to be as similar as possible across sites and scanners. Imaging analyses include segmentation and parcellation of structural data, diffusion measures, structural and functional connectivity analyses as well as MR spectroscopy

Of the 129 included study participants, MRI data are available for TP1 (*n* = 125), TP2 (*n* = 108), and TP3 (*n* = 100). 96 participants completed MRI at all three time points. Data quality has been continuously monitored and data has been preprocessed (Fig. 3). Moreover, diffusion imaging data has been harmonized to correct for between-site differences (Cetin Karayumak et al., 2019).

### Fluid biomarkers

Using of the miRNA arrays that allows for quantification of 179 different microRNAs in a single plasma sample we performed primary screening employing RT-qPCR technique. We have identified a population of miRNAs with altered expression in soccer players when compared to controls and also in the longitudinally collected samples in control and soccer players. So far we have identified several panels of deregulated miRNAs as primary hits for further investigation as potential diagnostic or prognostic biomarkers. After the validation of primary hits the data are prepared for bioinformatics and prediction of gene targets and deregulated molecular pathways associated with RHI.

### Data storage

The data were de-identified using an alphanumeric code without any personal information. Identifiable information is kept separate from de-identified research data. To ensure that data security is consistent with the European General Data Protection Regulation (GDPR), data is stored on secure servers while providing individual access to the REPIMPACT Consortium Investigators. A common Research Electronic Data Capture (REDCap) (Harris et al., 2009) database was created and maintained to ensure standardized and structured demographic and clinical data entries. Neuroimaging and balance data were stored on secure servers. Prior to uploading, data was stored locally in each site. Uploaded data was fully de-identified and neuroimaging data was “de-faced”. Following the upload, the neuroimaging data was organized according to Brain Image Data Structure (BIDS) (Gorgolewski et al., 2016) criteria. Blood and saliva samples were stored locally at −80 °C and after the end of data collection securely transferred to Slovakia for further analysis.

### Data Curation

Throughout the study, uniformity of data between sites and data quality have been monitored, discussed, and addressed in the monthly Consortium calls and annual Consortium meetings. Further, inclusion and exclusion criteria have been discussed whenever necessary. Following the completion of data collection, data have been digitalized using the secure web application REDCap. Consistency in entering the data has been ensured across all three sites. A master file was prepared that summarizes the completeness of data for all questionnaires and assessments and made available to all Consortium investigators. Moreover, any additional information (e.g., detailed information on medical history or data quality) that was deemed important for the interpretation of the data was prepared in order to share information. Quality checks have been performed for all questionnaires, clinical evaluations and neuroimaging data, and quality issues and artefacts have been noted and categorized.

## Challenges

### Between site inconsistencies

The difference in timing of the competitive season between the three data acquisition sites represented a challenge. While soccer teams in Germany and Belgium begin their season in late August of a given year, teams in Norway start in late January. Since, REPIMPACT aimed to include comparably competitive non-contact sports athletes, the difference in timing of the play season also affected the choice of non-contact sports: the Germany and Belgium sites included mostly swimmers as well as rowers and table tennis players while the Norway site also included sports that have their competitive season during winter time (e.g., cross-country skiing). These differences reflect national and regional differences between sites.

Differences in characteristics of control participants became apparent during data curation. Initially, exclusion criteria included participation in soccer beyond age 12 years. However, during the study it became evident that recruitment of control participants was challenging and that particularly participation in soccer was very common given the popularity of the sport across Europe. It was then decided to also include those who stopped participation in soccer at least 12 months prior to enrolment in REPIMPACT. In our database this group of controls has been labeled as “intermediate controls”. The proportion of controls and intermediate controls varies between study sites and this difference between sites will be taken into account when performing statistical analyses.

### Quantification of head impact exposure

REPIMPACT intended to measure head impact exposure using the in-ear sensor MV1 (MVTRAK, Durham, NC, USA). Thus, before the start of the cohort study, we performed both laboratory and on-field evaluation for the MV1 sensor, which demonstrated major challenges (Sandmo et al., 2019). In brief, in the laboratory setting, the sensor was mounted to a Hybrid III headform (HIII) and impacted with a linear impactor or football (range: 9-144 g). Random and systematic error were calculated using HIII as reference. The MV1 sensor showed considerable random error and substantially overestimated head impact exposure. While MV1 displayed accuracy in counting the number of head impacts, it provided inaccurate information on the magnitude of acceleration. Most importantly, due to poor positive predictive value for detecting headers in real-life settings, secondary verification would be needed, using e.g. video analysis or direct observation. Further, the substantial effort required for installing the devices on each individual player before each training and match made them infeasible to use. As a result of this experiment we decided not to use the MV1 sensor in the REPIMPACT study.

Instead of using physical sensors, REPIMPACT validated and applied a questionnaire known as HeadCount (Catenaccio et al., 2016), using self-reported head impacts as a measure for estimating periodical head impact exposure. The validation study (for a full report see Sandmo, Gooijers, et al., 2020b) was conducted at all three sites. In brief, we found that self-reported data could be used to group youth players into high and low heading exposure groups, but not to estimate individual heading exposure. We then used this questionnaire to capture information on head impacts experienced by our study participants. More accurate methods for estimation of head impact exposure remain an important challenge for the field.

### Neuroimaging

During the first year of the study, a hardware failure made an update of the MR scanner at the German site necessary. Following this update, the image quality of the multi-band diffusion and resting-state fMRI sequences was insufficient. The failure was identified through periodic quality control of the obtained images. Following the identification of the issue it was decided to no longer use the multi-band option in the German site, and instead, the non-multi band sequence from Norway was installed and tested at the German site. To account for this change in sequences in the image processing, harmonization and statistical analyses we defined the German data as originating from two sites, one with multi-band data, and one without.

## Conclusions

REPIMPACT aims to comprehensively address the gap in knowledge regarding the effects of RHI on brain structure, function, biochemistry and development in competitive youth soccer players. Between 2017 and 2020, REPIMPACT enrolled 129 youth athletes in three European countries employing a multimodal and multidimensional approach of comprehensive measures across many domains. The study includes advanced neuroimaging techniques including novel, sensitive, and specific measures that are expected to be better associated with clinical and behavioral outcome measures. As such, the REPIMPACT study is positioned to address several key scientific questions regarding the effects of exposure to RHI on the brain. The Consortium proactively applied several measures to ensure consistency of participant inclusion, data collection, and data quality across the data acquisition sites, yet, there is the possibility of site-specific effects in the assessment of study participants. As with most multisite multinational studies of this scope, there were challenges and limitations (e.g., only male youth athletes were included). By reporting the setup of the study, the limitations, challenges and how we addressed them, we intend this manuscript to be of use for researchers that are planning to establish new multisite studies.

In summary, the REPIMPACT Consortium is on track to making important contributions to our understanding of the effects of RHI on the brain in youth soccer athletes. Moreover, we anticipate that this study will pave the way for management guidelines and ultimately for prevention of brain alterations due to exposure to RHI in athletes.

## Data Availability

All data and material used are available upon request.

## Declarations

### Consortium members

Michaela Bonfert, Elena M. Bonke, Sylvain Bouix, Fanny Dégeilh, Alexandra Gersing, Malo Gaubert, Felicitas Heinen, Florian Heinen, Stefan Hillmann, Leonard Jung, David Kaufmann, Elisabeth Kaufmann, Janna Kochsiek, Marc Muehlmann, Paul S. Raffelhueschen, Paula C.M. Schorlemer, Bettina Schwarz-Moertl, Johanna Seitz-Holland, Alexandra A. C. Silva, Uta Tacke, Lisa Umminger, Tim L.T. Wiegand, Caroline Seer, Amanda Clauwaert, Thiago S. Monteiro, Alberto de Luca, Doron Elad, Stian Bahr Sandmo, Thor Einar Andersen, Erling Hisdal, Audun Holm Torgersen, Martin Cente, Igor Jurisica, Katarina Matyasova, Sara Porubska and Jozef Hanes.
